# Vaccine Acceptance During a Novel Student-led Emergency Department COVID-19 Vaccination Program

**DOI:** 10.5811/westjem.58728

**Published:** 2023-05-05

**Authors:** Carly Eastin, Brendan Moore, Aaron Moulton, Luke Lefler, Fuad Haydar, Morgan Sweere, Gavin Jones, Crystal Sparks, Austin Porter, M. Kathryn Allison, Travis Eastin

**Affiliations:** * University of Arkansas for Medical Sciences, College of Medicine, Department of Emergency Medicine, Little Rock, Arkansas; † University of Arkansas for Medical Sciences, College of Medicine, Little Rock, Arkansas; ‡ University of Florida, College of Medicine, Department of Emergency Medicine, Jacksonville, Florida; § University of Arkansas for Medical Sciences, Department of Pharmacy and Therapeutics, and College of Medicine, Department of Emergency Medicine, Little Rock, Arkansas; || University of Arkansas for Medical Sciences, College of Public Health, Department of Health Policy and Management; Arkansas Department of Health, Little Rock, Arkansas; # University of Arkansas for Medical Sciences, College of Public Health, Department of Health Behavior and Health Education, Little Rock, Arkansas

## Abstract

**Introduction:**

The coronavirus 2019 (COVID-19) pandemic not only exacerbated barriers to healthcare but has also highlighted the trend toward increased vaccine hesitancy. Our goal was to improve COVID-19 vaccine uptake through a student-led, emergency department-based (ED) vaccination program.

**Methods:**

This prospective, quality-improvement pilot program used medical and pharmacy student volunteers as COVID-19 vaccine screeners in a southern, urban, academic ED. Patients eligible for vaccination were offered either the Janssen-Johnson & Johnson or the Pfizer-BioNTech COVID-19 vaccine and were educated about vaccine concerns. Vaccine acceptance rates were recorded, as well as reasons for vaccine hesitancy, vaccine brand preferences, and demographics. The primary and secondary quantitative outcomes were overall vaccine acceptance and change in vaccine acceptance after student-provided education, respectively. We performed logistic regression to identify potential variables that correlated with vaccine acceptance. Guided by the Consolidated Framework for Implementation Research, focus group interviews with four key stakeholder groups explored implementation facilitators and barriers.

**Results:**

We screened 406 patients for COVID-19 vaccination eligibility and current vaccine status, the majority of whom were unvaccinated. Of unvaccinated or partially vaccinated patients, vaccine acceptance before education was 28.3% (81/286), and vaccine acceptance after education was 31.5% (90/286) (% difference, 3.1% [95% CI 0.3%–5.9%], P=0.03). The most common hesitancy factors cited were concerns about side effects and safety. Results from the regression analysis indicated that increasing age and Black race were associated with an increased odds of vaccine acceptance. Focus groups revealed implementation barriers, including patient resistance and workflow issues, and facilitators, including student involvement and public health promotion.

**Conclusion:**

Using medical and pharmacy student volunteers as COVID-19 vaccine screeners was successful, and brief education provided by the students led to a modest increase in vaccine acceptance, with overall acceptance of 31.5%. Numerous educational benefits are described.

## INTRODUCTION

Severe acute respiratory syndrome coronavirus, the virus that causes coronavirus 2019 (COVID-19), was first identified in 2019 but quickly spread globally, leading the World Health Organization to declare a worldwide pandemic in March 2020.^1^ Widespread vaccination has been a crucial aspect of the public health response to the COVID-19 pandemic, contributing to the generation of immunity in the general population.^2^ The available COVID-19 vaccines are highly effective—decreasing symptomatology, transmission, hospitalization, and death.^3^

A significant challenge to vaccination is vaccine hesitancy, defined as delay or refusal of vaccination despite availability,^4^ which has been increasing over the past two decades.^5^ Vaccine hesitancy is pervasive among emergency department (ED) patients and can diminish their trust in informational sources regarding vaccines.^2^,^6^ Among unvaccinated individuals, data suggests decreased trust in medical professionals and medical care. Studies in Arkansas, where the current study took place, demonstrated relatively low rates of vaccination and high rates of vaccine hesitancy.^7^,^8^

The ED serves as the primary healthcare resource for approximately one-fifth of the United States (US) population. These underserved patients have been disproportionately affected by the COVID-19 pandemic and are a prime target for a public health response.^9^ Studies suggest that ED-based COVID-19 vaccine interventions may be a way to reach these vulnerable populations, similar to other public health interventions (eg, HIV testing and influenza vaccination).^9^–^11^

The purpose of this quality improvement (QI) pilot study was to improve vaccine uptake among patients offered COVID-19 vaccination in the ED. While it can be difficult for the care team to have thoughtful conversations with vaccine-hesitant patients due to time constraints, other resources such as students^12^,^13^ may be available. Therefore, in this study we examine COVID-19 vaccine acceptance using medical and pharmacy students as dedicated COVID-19 vaccine screeners for ED patients.

## METHODS

### Study Design and Setting

We conducted a prospective, observational QI pilot in a single, adult, tertiary care, inner-city ED with an annual volume of approximately 60,000 patients. There were two phases: Phase 1 occurred May 21–June 6, 2021, and Phase 2 June 28–August 31, 2021. We chose these dates due to the availability of medical and pharmacy students during their summer break.

Based on a Plan-Do-Study-Act model, Phase 1 served as a needs assessment to measure potential vaccine acceptance.^14^ In this phase, ED patients were screened for their interest in COVID-19 vaccination. Once we identified sufficient interest in ED-administered vaccines, predefined as theoretical vaccine acceptance of ≥10%, we transitioned to Phase 2 by offering and administering both Janssen-Johnson & Johnson (stocked in the ED) and Pfizer-BioNTech COVID-19 vaccines (stocked in inpatient pharmacy) to ED patients. At the time, the Moderna COVID-19 vaccine was not on formulary. After two months of Phase 2, we conducted a qualitative study of the process through focus-group interviews of key stakeholders.

**Population Health Research Capsule**

What do we already know about this issue?

*Vaccine hesitancy has been a growing public health concern exacerbated by the recent COVID-19 pandemic. As a result, vaccine uptake has been suboptimal*.

What was the research question?

*Can a student-led, ED-based COVID-19 vaccine program adequately address vaccine concerns and improve uptake?*

What was the major finding of the study?

*Medical student education of patients in the ED increased vaccine acceptance rates from 28.3% to 31.5% (mean difference 3.1%, 95% CI 0.3%-5.9%, P=0.03)*.

How does this improve population health?

*A student-led COVID-19 vaccination program can successfully provide patient education and facilitate vaccine uptake in the ED setting, potentially reducing the burden of this disease*.

Because this vaccine initiative was part of a QI project, the university institutional review board determined that this was not human subjects research. We followed the Revised Standards for Quality Improvement Reporting Excellence (SQUIRE)^15^ reporting guidelines where appropriate.

### Selection of Participants

Patients aged ≥12 years were included based on the Emergency Use Authorization (EUA) approval during the study period. Patients <18 years were required to have a guardian present. See Table 1 for details regarding included patients.

Medical and pharmacy students approached patients in four-hour shifts between 10 am and 10 pm daily, with weekdays prioritized. Shifts were shortened to two hours later in the study when the students’ fall classes started. In both phases, the screening process consisted of students assessing for eligibility using the ED trackboard. Early in the initiative, once a student found a patient meeting the age restrictions who was not dispositioned to be admitted, they would approach the patient. In mid-June 2021, our electronic health record (EHR) update released a banner in every chart that alerted the treatment team to the patient’s COVID-19 vaccination status, which was synced with the health department’s statewide vaccine database. From this point forward, students would enter the chart and assess the vaccine status for patients who fit the age criteria who were not dispositioned to be admitted. If the patients were noted to be fully vaccinated, they were excluded. Partially vaccinated and unvaccinated patients were approached and further assessed for eligibility.

At the start of their shift, students would begin screening in the main ED and the ED clinical decision unit, an ED observation unit that holds observation patients or ED overflow patients. After they had screened and assessed all roomed ED patients, they would check for any new roomed patients before moving to the waiting room or triage area to approach patients waiting to be seen. Once a patient had been approached, patients were not questioned again during that ED visit unless the encounter had been interrupted and not yet completed or the patient asked for time to consider their response. Patients receiving active medical care could be reapproached later in the visit if that portion of their care had been completed and they were available for questioning, but they were excluded if still receiving active medical care or were critically ill.

Overall, 39 students participated, many working in pairs. Before approaching patients independently, students were required to review educational materials on the available COVID-19 vaccines and complete an in-person orientation, which included supervised patient encounters. Students were also provided with a script to facilitate discussion (see Appendix 1).

### Intervention

For vaccine-eligible patients, student volunteers offered the vaccine, recorded concerns, provided education, and then offered the vaccine again. Based on a previous study on vaccine hesitancy, patients’ concerns were categorized as follows: efficacy; safety; side effects; belief that it was unnecessary; belief it was not needed due to prior COVID-19 infection; cost or financial concerns; other, or no concerns.^16^ If the patient had questions or concerns that the student could not adequately address, the student notified the clinician(s) caring for the patient.

Before vaccines were available in the ED (Phase 1), accepting patients were scheduled at a COVID-19 vaccine clinic. In Phase 2, patients vaccinated in the ED received the required US Centers for Disease Control and Prevention (CDC) COVID-19 vaccine card and applicable Emergency Use Authorization (EUA) fact sheet, as well as information about when and where to obtain a second dose, if indicated. Scheduling in the vaccine clinic was still an option if patients were accepting but declined to be vaccinated in the ED. For patients needing to complete a two-shot series, the second dose was given based on the CDC guidelines. Booster shots were not in use at the time of this study. Due to EHR limitations and hospital policies, only the bedside nurse or a paramedic could physically administer the vaccine. During Phase 2, the ED care team could administer vaccines as part of routine care when student volunteers were not available; however, data on those patients was not collected.

### Quantitative Data Collection

Data was recorded using a departmental iPad (Apple Inc, Cupertino, CA). For each patient interviewed, we recorded vaccination status, contraindications to COVID-19 vaccination, vaccine acceptance before and after education, and demographic information related to gender identity, race, and ethnicity. Age was recorded from the EHR. After the initiative, we used a pharmacy report of all patients who were vaccinated in the ED to confirm whether patients recorded in our study as accepting of the vaccine were vaccinated and to quantify the number of vaccines administered outside student volunteer hours.

### Qualitative Data Collection

We used the Consolidated Framework for Implementation Research (CFIR)^17^ to guide qualitative data collection and analysis. We developed a semi-structured interview guide to explore CFIR constructs as potential influences on COVID-19 vaccine implementation in the ED. After two months of the administration phase, the study team (TE, CE, BM, and AM) conducted semi-structured focus group interviews with key stakeholders, which included 12 medical students, 15 emergency medicine (EM) resident physicians, and 10 EM faculty physicians. Due to difficulty with scheduling a focus group of nurses, we approached ED nurses at random for one-on-one interviews, resulting in 10 interviews with ED nurses. In total, 47 individuals participated in a qualitative interview or focus group. We used a core set of questions for all stakeholder groups, with the addition of specific questions tailored to each stakeholder group (see Appendix 2). All interviews were audio recorded and then transcribed.

### Outcomes

For analysis, we combined both phases. The primary outcome was vaccine acceptance after education among vaccine-eligible patients. The secondary outcome was the change in vaccine acceptance after education was provided by the students. Common causes of COVID-19 vaccine hesitancy were reported. We performed a post-hoc analysis to compare the number of vaccines given per student hour vs non-student hour. Lastly, we describe implementation facilitators and barriers, educational impacts, and recommendations to improve future implementation processes identified from focus-group interviews.

### Analysis

We used descriptive statistics for demographic data. Vaccine acceptance was defined as answering “Yes” when offered the vaccine; vaccine refusal was defined as either answering “No” or “Unsure.” Because we were comparing vaccine acceptance rates within the same population before and after education, a paired sample *t*-test was used to compare proportions of vaccine acceptance. We used chi-squared testing to compare rates of vaccine administration between student-covered hours and uncovered hours. Logistic regression was performed to determine whether any factors were predictive of vaccine acceptance, such as age, gender, race, ethnicity, or category of vaccine-related concerns. All comparisons were made using a two-sided approach with ɑ = 0.05, and 95% confidence intervals are reported where appropriate. Cases that were eligible for vaccination but had missing outcome data were treated with case deletion. If only demographic responses were missing, these cases were included. Data were entered into REDCap, a research electronic data capture tool hosted at the University of Arkansas for Medical Sciences and analyzed in SPSS Statistics for Macintosh version 28.0 (IBM Corp, Armonk, NY) and SAS version 9.4 (SAS Institute, Inc, Cary, NC).

The CFIR was used to guide qualitative analysis. Using an inductive approach, we performed thematic analysis of the transcribed interviews, starting with individual coding by authors (TE, CE, BM, and AM).^17^ After individual coding, the authors (TE, CE, BM, AM, and MS) met to compare and discuss individual coding. Based on group consensus, coding was revised and organized into major themes.

## RESULTS

### Quantitative Results

**Figure 1:**
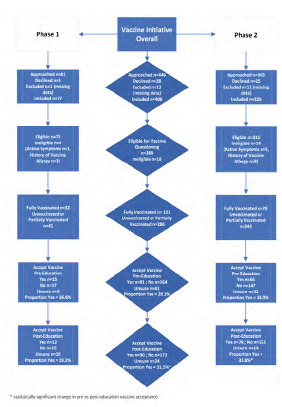
Flowchart of all patients approached and enrolled in emergency department program of medical student education to promote COVID-19 vaccination.

We transitioned to Phase 2 after 16 hours of Phase 1, as we met our predetermined threshold for theoretical acceptance of 10% (Phase 1 acceptance rate 29.2%). Combining both phases, we analyzed 406 patients. The average age was 43.5 years (SD 16.3), and the majority were female (55.3%) and reported being Black (50.8%). See Figure 1 for a detailed patient flow chart and Table 2 for demographic information. Of the 388 patients eligible for vaccine questioning, 26.2% were already fully vaccinated. Before education, 286 patients were offered the COVID-19 vaccine, with 81 accepting (28.3%), 164 declining (57.3%), and 41 (14.3%) unsure. After education, 90 agreed to be vaccinated (31.5%); 172 declined (60.1%), and 24 were unsure (8.4%). The change in vaccine acceptance after education was statistically significant (Table 3). The most common vaccine-related concerns were regarding side effects (26.9%), safety (22.4%), or other (11.9%), while many had no concerns (40.2%).

Regression analysis revealed that Black patients were associated with a near three-fold increase in the odds of vaccine acceptance when compared to White patients (OR 2.7, 95% CI 1.30–5.59; *P*=0.008). We also found that every year increase in age was associated with a 3% increase in the odds of vaccine acceptance (OR 1.03, 95% CI 1.00–1.05; *P*=0.005). Additionally, patients who stated they “did not believe the vaccine was necessary” or had “other concerns” were significantly less likely to be vaccinated (Table 4).

Based on pharmacy data during Phase 2, 68 of 78 patients (87.2%) who accepted the vaccine were vaccinated. Of the remaining 10 patients, one needed Moderna which was unavailable, two received their vaccinations within 30 days of the ED visit, and one patient had a vaccine ordered but then discontinued. Details on the remaining patients were not available.

The students covered 140 ED hours during Phase 2. This left 1,420 ED hours without coverage, during which an additional 85 patients were vaccinated as part of routine care. Based on these confirmed administrations, there were 0.49 vaccinations per student hour vs 0.06 vaccinations per non-student hour, indicating a significant difference in vaccination during times with student coverage (relative risk 8.1, 95% CI 6.2–10.6, *P*<0.001).

### Qualitative Results

The medical students, EM nurses, residents, and faculty involved provided valuable insight into their experiences, revealing 1) barriers to implementation; 2) facilitators to implementation; 3) educational impacts; and 4) recommendations for process improvement (Table 5). We used the CFIR to analyze and describe barriers and facilitators to implementation.^17^

#### Barriers to Implementation

##### Patients’ Needs and Resources

Many of those interviewed said that patients were resistant to the vaccine. They perceived that the patients’ established religious and political beliefs and opinions about the safety and efficacy of the vaccine contributed to this resistance. Some participants said that patients expressed various safety concerns, such as risk of thromboembolic events. One nurse cited instances where patients became angry and “political.” Similarly, a resident physician referred to vaccines as a “hot topic,” causing the patient to be “mad the rest of the visit,” while a faculty physician reported that mentioning the vaccine “made the patient upset.”

##### Compatibility with Existing Workflow

Participants described the screening and vaccine administration process and its incompatibility with ED workflows as a barrier to implementation. Most patients preferred the Pfizer-BioNTech vaccine, which had to be retrieved in person by walking to the inpatient pharmacy; participants stated that obtaining this vaccine was “time consuming” and “cumbersome.” One nurse also reported that the screening questions (when student screeners were not available) were “another thing to tack on” to the existing triage process. Multiple faculty physicians referenced workflow interruptions and increased length of stay. Some students said that the vaccine screening impeded the nursing triage process. One student said, “I felt in the way in triage.”

#### Facilitators to Implementation

##### Patients’ Needs and Resources

Participants described a common goal of improving the health of patients on an individual and population level, which facilitated implementation of this project. One faculty member said, “Sometimes it feels like it is the biggest thing I accomplish in a shift.” Another faculty member felt that they were doing their part to promote public health and “reduce burden of illness.” Participants, particularly nurses, expressed their belief that the project increased access to the vaccine among patients who might otherwise have significant barriers to receiving healthcare, such as lack of transportation. One nurse stated that “exposure is key, especially in people that wouldn’t have [access] otherwise.” Nearly all participants felt the project was “worthwhile” and should be continued.

##### Available Resources

Multiple nurses reported that it was far easier to implement the vaccine protocol when students were present. Physicians felt they did not have time to approach patients directly to offer the vaccine, but the students’ presence both reminded them and allowed them to delegate that time-consuming task. Physicians also said that the students had “plenty of time” to do the screening.

##### Implementation Process

Similarly, participants noticed increased vaccine uptake when the screening process was started early in the ED visit and that workflow was improved when the process was initiated during triage. Participants felt that this gave the patient time to consider the vaccine and limited delays at discharge. Additionally, students felt that having a script facilitated implementation.

#### Educational Impacts

The perceived educational benefits to the students were clear across multiple interviews. One resident noted that they “seemed very excited” to be involved. Students cited patient interaction, experience having difficult conversations, exposure to the clinical environment, and EHR experience as educational benefits. One student said, “It was a good skill to learn how to react when talking to … patients who didn’t want the vaccine.”

Students appreciated the clinical experience in light of curricular changes due to the COVID-19 pandemic. One student said, “I haven’t gotten to spend a lot of time in the hospital because of the pandemic, so just getting to talk to patients one on one was really helpful.” Another student said, “I thought it was also helpful to interact with [the EHR] and learn to utilize it and put in an order.”

Most students reported positive experiences interacting with ED staff, describing residents as “helpful,” “excited,” and “complimentary.” Some students felt that similar projects should be offered permanently as elective courses. The educational value was not limited to students. One resident physician reported, “I know more information about the COVID-19 vaccines because of this. Reading more about the data and literature for the vaccines than I probably would have done.”

#### Recommendations for Process Improvement

Every group recommended ways to improve the implementation process. A common suggestion was to expand the role of students to include vaccine administration. Multiple nurses and students felt that allowing students to administer vaccines would have significantly improved the workflow. Participants also recommended that COVID-19 vaccination be discussed with the patient early in their ED stay to avoid delays. Participants recommended improving educational materials and resources related to vaccination, such as providing a handout of vaccine statistics or other background information for students to reference, as well as playing informative video messages or having an educational poster in the waiting room. A few staff reported that they did not feel well trained in the COVID-19 vaccine screening and administration process and that additional instruction would have been helpful.

## DISCUSSION

The COVID-19 pandemic presented unique challenges for ED patients, clinicians, and even students in clinical training. Because our ED reaches underserved groups, we had the opportunity to assist populations with less access to the COVID-19 vaccine. In an effort to increase vaccination rates within our patient population, we piloted a student-facilitated, ED-based COVID-19 vaccination campaign.

Overall, our pilot campaign was successful in increasing vaccine uptake through a novel student-learning experience. In addition to scheduling patients in our institution’s vaccine clinic, we vaccinated 68 patients in the ED in two months using student-led patient screening and education. Past studies have shown the value of involving medical and pharmacy students in influenza vaccination initiatives,^12^,^13^,^18^ but to our knowledge, studies about student-facilitated, ED-based COVID-19 vaccination programs are lacking. We found a higher vaccine acceptance rate after a brief educational intervention and that more vaccines were given when students were present.

Interestingly, in Phase 1 we saw a non-significant downward trend in vaccine acceptance after the educational intervention. Possible explanations for this include level of student experience and comfort in providing education early in the initiative, lack of effectiveness of the education, or simply the small sample size in Phase 1. Fortunately, we found a statistically significant increase in post-intervention acceptance in Phase 2 and in the pooled data. Even if this small overall increase based on education may not be clinically significant, we feel that given the reduction in morbidity and mortality risk associated with vaccination that every additional vaccinated patient is beneficial. Regardless of the educational intervention, having students available in the ED to offer vaccines considerably increased our chances of vaccination. We feel this adequately shows that medical and pharmacy students can effectively screen and educate patients about vaccine safety, efficacy, and concerns. While the typical nurse or physician in the ED may not have time to counsel patients on COVID-19 vaccine concerns, student volunteers can fill this role effectively.

Despite the successful vaccination of many patients, the COVID-19 vaccine campaign had multiple barriers to implementation in the ED, which our study identified. The most significant barrier was vaccine hesitancy, as only 31.5% of vaccine-eligible patients accepted the vaccine. Common hesitancy factors were concern for safety, side effects, efficacy, feeling it was not necessary, and several other specific concerns. These mirror barriers encountered in recent studies on influenza and COVID-19 vaccine hesitancy in the ED.^2^,^9^,^19^,^20^ The modest increase in vaccine acceptance after a brief educational intervention may suggest underlying unmet educational needs or the need for additional information, which is similar to findings by Rodriguez et al, who found lack of information was a common contributor to hesitancy.^9^

However, patients’ beliefs that vaccination was unnecessary predicted lower acceptance, indicating that overcoming preconceived sentiments about vaccine necessity may be difficult. Likewise, Willis et al found that low fear of COVID-19 predicted lower acceptance.^8^ We also found that patients with “other” concerns were less likely to be vaccinated, possibly because they had very specific concerns that we could not adequately address. We suspect that these factors most contributed to our difficulty in achieving a more robust change in post-education acceptance; some patients were open to the discussion, but most who declined felt it simply was not needed and were not interested in hearing about the risks of COVID-19 infection or the benefits of vaccination.

Other non-patient related barriers included interference with ED workflow and increased workload for ED staff. These barriers are congruent with studies on influenza vaccination, which found that nurses believed that an ED-based vaccination program was “too time consuming” and cited a “need to simplify documentation process.”^19^ We received similar feedback; having students serve as screeners helped to reduce these negative impacts.

Mitigating workflow barriers was an important part of facilitating the implementation of our program. This included introducing COVID-19 vaccination early in a patient’s ED visit (when possible), having dedicated individuals to screen, and providing training and a script to students. Cohen et al also found that having dedicated staff (pharmacists) providing the screening and counseling for influenza vaccinations improved the feasibility of ED-based vaccinations.^21^ Similar to other community-based vaccination programs,^18^ the desire of stakeholders to provide a worthwhile public health initiative was a strong motivator, as was helping to eliminate barriers for underserved patients, such as transportation.

Our study explored associations between vaccine acceptance and demographic characteristics. Prior literature reveals mixed findings on the association between age and COVID-19 vaccine acceptance.^2^,^9^,^16^,^22^ Similar to another study in Arkansas, we found that increased age correlated with vaccine acceptance, although this association was not particularly strong.^8^ In regard to race, recent studies have shown that Black Americans are less likely to accept the COVID-19 vaccine, more likely to delay vaccination, and more likely to report mistrust of the vaccine when compared to White Americans.^8^,^23^ However, our study found that Black patients were more likely to accept the COVID-19 vaccine. A recent qualitative study of 72 Black and Latinx individuals revealed the influence of distrust in COVID-19 acceptance, as well as the importance of providing consistent, fact-based information to inform trust and addressing structural barriers to vaccination.^24^ Although this was not the focus of our study, it is possible that we saw higher vaccine acceptability among this group because the program made the vaccine more readily available and provided fact-based education.

The COVID-19 pandemic affected not only patients and frontline healthcare workers but also disrupted medical education. In-person clinical rotations were often replaced with virtual learning experiences, and many medical students perceived this lack of clinical experience as a lapse in their medical education.^25^ Fortunately, once our institution allowed students back into the clinical setting, our program provided a new opportunity for clinical experience while also reducing the burden of vaccination on clinical ED staff. While there have been student-led vaccine campaigns in other settings, to our knowledge this was the first ED-based, student-facilitated vaccination program. The students cited many positive educational impacts, including clinical exposure in the time of COVID-19 when such exposure was lacking, an introduction to having difficult conversations with patients, and the development of skills for educating their future patients about the importance of vaccines as part of preventive health. These clinical, knowledge, and communication benefits were similar to those found in other student-led influenza-vaccine initiatives, and we feel these educational benefits will continue even when clinical rotations are not restricted.^18^

While previous ED-based studies reported COVID-19 vaccine acceptance of 50–70%,^2^,^9^ acceptance in our study was only 31.5%, with 23.8% having confirmed inoculations. We have two potential explanations for this variance. First, our study occurred in summer 2021 after COVID-19 vaccines were widely available and only those eligible for vaccination were analyzed, missing those who had already received it. Second, we were giving vaccines for most of our study, while the prior studies examined hypothetical acceptance. Farrell et al (2022) and Ford et al’s (2022) preliminary data on automated, EHR-prompted, ED-based COVID-19 vaccination programs suggest a much lower true acceptance rate (2.6% in all unvaccinated patients,^26^ and 3.6% in homeless patients targeted,^27^ respectively). Our results are more consistent with Cohen et al who found an acceptance rate of 41% when initiating pharmacist-driven, ED influenza vaccinations, possibly due to both our programs having an approach based on personal interaction and education, rather than being computerized.^21^

### Implications and Recommendations for Future Intervention

This program demonstrated that medical and pharmacy students can be an invaluable resource in spearheading ED vaccination campaigns while participating in a valuable educational experience. We believe that our framework could be used to develop other student-driven, preventive health programs implemented in EDs. Our next goal is to finalize a formal ED-based public health elective rotation, during which students can continue this important work, while receiving credit toward their degrees.

## LIMITATIONS

There were several limitations. First, this work was performed at a single site. While many EDs likely share similar barriers or facilitators to an ED-based vaccination program, our findings may not be universally applicable and patient populations of other sites may differ. While we did survey patients on their opinions about potential hesitancy factors, our qualitative data does not include patient perspectives. Additionally, although both pharmacy and medical students were vaccine screeners in this project, we only interviewed medical students about their experiences; pharmacy students may have had different perspectives. We were also limited by the short pilot period and small sample size due to resumption of student classes. Response bias may have introduced a trend toward vaccine acceptance, which may be reflected in the findings that only 86% of patients in Phase 2 who accepted were vaccinated in the ED; some likely changed their mind when the vaccine was ready to be given. Finally, outside of quantifying vaccinations, we were not able to collect data on patients who were offered vaccination outside student volunteer hours and specifics on patients scheduled in the vaccine clinic were not available.

## CONCLUSION

Emergency departments within academic health centers are ideal environments in which to disseminate the COVID-19 vaccine to underserved patients, as well as engage healthcare students in vaccine screening. Our pilot study found that nearly one-third of patients were willing to be vaccinated, and patients were slightly more likely to accept the vaccine after a brief educational intervention. This student-led model is unique as both healthcare students and patients benefited from the educational component of the vaccine campaign. Although patients’ concerns about the vaccine and workflow interruptions were implementation barriers, facilitators included the involvement of students, providing scripts for students, and clinicians’ perception that the initiative improves patients’ access to the vaccine. Our study suggests that a student-led, COVID-19 vaccine initiative is not only feasible in the ED but viewed as promoting public health and providing a valuable educational experience.

## Supplementary Information





## Figures and Tables

**Table 1:** Inclusion and exclusion criteria for patients offered a COVID-19 vaccination in the emergency department.

Inclusion Criteria ED patients ≥12 years old Included but ineligible for vaccination Contraindication to COVID-19 vaccination (e.g., history of vaccine allergy) Already fully vaccinated by self-reporting Had COVID-19 test pending or active symptoms related to COVID-19 Exclusion Criteria <18 years old without a guardian Already fully vaccinated based on notification in electronic health record Dispositioned to be admitted^a^ Undergoing active medical care (e.g., clinician in room, patient undergoing a procedure or test/imaging) In respiratory isolation Patient declined to participate

a Admitted patients not included because they were offered vaccination at inpatient discharge.

*ED*, emergency department; *COVID-19*, coronavirus 2019.

**Table 2:** Patient demographics, history of vaccination, and and vaccine hesitancy characteristics.

		All (N=406)		Study Phase			
				Phase 1 (N=77)		Phase 2 (N=329)	
		N	%	N	%	N	%
Age (years), mean, SD		43.5	16.3	48.4	17.1	42.45	15.9
Gender^a^	Male	166	44.7%	19	37.3%	147	44.7%
	Female	205	55.3%	32	62.7	173	54.1%
Race^a^	Black	188	50.8%	30	58.8%	158	49.5%
	White	139	37.6%	20	39.2%	119	37.3%
	Hispanic	25	6.8%	1	2.0%	24	7.5%
	Asian	1	0.3%	0	0.0%	1	0.3%
	American Indian/Alaska Native	2	0.5%	0	0.0%	2	0.6%
	Native Hawaiian/Other Pacific Islander	0	0.0%	0	0.0%	0	0.0%
	Multiple races	15	4.1%	0	0.0%	15	4.7%
Ethnicity^a^	Hispanic or Latino	36	9.8%	3	5.9%	33	10.4%
	Not Hispanic or Latino	333	90.2%	48	94.1%	287	89.6%
History of COVID-19 Vaccination (N=388)	Yes with 2-shot series	83	21.4%	29	39.7%	54	17.1%
	Yes with single shot (Janssen–Johnson & Johnson)	19	4.9%	3	4.1%	16	5.1%
	Scheduled but not yet received	2	0.5%	1	1.4%	1	0.3%
	First dose received, second scheduled	8	2.1%	0	0.0%	8	2.5%
	First dose received, second not scheduled	12	3.1%	1	1.4%	11	3.5%
	No vaccine	264	68.0%	39	53.4%	225	71.4%
Vaccine Concern (N=388)	Efficacy	19	6.6%	0	0.0%	19	7.6%
	Safety	64	22.4%	7	17.1%	57	22.9%
	Side effects	77	26.9%	14	34.1%	63	25.3%
	Do not believe it is necessary	22	7.7%	4	9.8%	18	7.2%
	Already had Covid-19	3	1.0%	0	0.0%	3	1.2%
	Cost/financial concerns	1	0.3%	0	0.0%	1	0.4%
	Other	34	11.9%	6	14.6%	28	11.2%
	No concerns or questions	115	40.2%	19	46.3%	96	38.6%
Preferred Vaccine	Do not want one	2	2.3%	0	0.0%	2	2.6%
	Pfizer-BioNTech	52	59.1%	9	75.0%	43	56.6%
	Janssen–Johnson & Johnson	33	37.5%	2	16.7%	31	40.8%
	No preference	1	1.1%	1	8.3%	0	0.0%

Data reported in n with proportions unless otherwise noted.

a Age recorded from electronic health record; other demographic data self-reported at the end of interview; some demographic responses missing.

*COVID-19*, coronavirus disease 2019.

**Table 3:** Change in vaccine acceptance after education: all vaccine-eligible patients.

Vaccine acceptance before education (N, % Yes)		Vaccine acceptance after education (N, % Yes)		Change in vaccine acceptance with education % difference (95% CI)
81/286	28.3%	90/286	31.5%	3.1% (0.3%–6.0%), P=0.03

*CI*, confidence interval.

**Table 4:** Logistic regression for primary outcome of vaccine acceptance

Variable	Adjusted OR (95% CI)	P-value
Age per year	1.03 (1.00, 1.05)	0.005
Male (ref=Female)	1.53 (0.84, 2.79)	0.17
Race^a^		
Black (ref=White)	2.70 (1.30, 5.59)	0.008
Other (ref=White)	2.08 (0.54, 8.00)	0.29
Hispanic (ref=non-Hispanic)	1.85 (0.45, 7.56)	0.39
Hesitancy^b^		
Efficacy	0.39 (0.08, 1.97)	0.25
Safety	0.48 (0.18, 1.31)	0.15
Side effects	0.49 (0.19, 1.28)	0.14
Do not believe it is necessary	0.08 (0.01, 0.73)	0.02
Other	0.21 (0.05, 0.84)	0.03
No questions	1.01 (0.38, 2.73)	0.98

a Racial categories were divided into Black, White, and other due to low prevalence of some races.

b Hesitancy categories of “already had COVID-19” and “cost/financial concerns” were removed from the regression model as they were rarely cited.

*OR*, odds ratio.

**Table 5:** Qualitative themes, CFIR* constructs, and theme descriptions

Theme	CFIR construct	Theme description	
Barriers to implementation	Patients’ needs and resources	Patients’ resistance to getting the vaccine Patients’ concerns for safety and side effects of the vaccine	Medical student: “[Hesitant patients] probably made up their mind beforehand.”
	Compatibility with existing workflow	Workflow interruptions Location of the preferred vaccines (Pfizer-BioNTech COVID-19 vaccines stored outside the ED at the hospital pharmacy)	Medical student: “Sometimes I felt like [the nurses] were so busy, they wondered why we couldn’t [administer the vaccine] ourselves. Especially in triage. It felt like we were adding a burden to the nurses.”
Facilitators to implementation	Patients’ needs and resources	Helping patients and promoting public health Improved access to care for patients who may otherwise not have access to the vaccine	Nurse: “Helping people that don’t have access to healthcare.” Physician: “Performing [our] civic duty”
	Available resources	Involvement and availability of students to screen patients	Resident physician: “Having the students there was great because they had more time to sit down and go over questions.”
	Implementation process	Early timing of screening in ED visit Scripting	Medical student: “The script was really helpful, if you went blank, to lean back on for every conversation.”
Educational impacts		Clinical experience for students during COVID-19 pandemic Improved student comfort with patient interactions, including having difficult conversations with patients	Medical student: “I think it has helped me learn to talk to patients and talk to them about something difficult and teach them something that could actually help their health. …it will make me a better doctor in the future learning how to talk to all different kinds of people.” Medical student: “I think we have all asked ourselves how we can help during the pandemic, and I think this is a really easy way to help, and you feel like you actually contributed something.”
Recommendations for process improvement		Expand the role of students (have students administer the vaccine) Workflow/early timing of introducing vaccine Improve education materials (eg, fact sheets, videos) Train staff on the screening and vaccine administration process	Medical student: “It would have been really nice if the students would have been able to give the shots because most of us are trained in that.” Nurse: “[The provided Vaccine Card and EUA Fact Sheet] don’t have all the answers. We can’t answer all their questions and don’t have the time.”

* *CFIR*, Consolidated Framework for Implementation Research; *ED*, emergency department; *EUA*, Emergency Use Authorization.
