# Supplementary material for: Vaccine Acceptance During a Novel Student-led Emergency Department COVID-19 Vaccination Program
Source: West J Emerg Med. 2023 May 5;24(3):436–46. doi: 10.5811/westjem.58728 (PMC10284509; doi:10.5811/westjem.58728)
Supplement: Supplementary file 1 [file wjem-24-436-s001.docx]

**Appendix 1: Student Script for Patient Interviews**

1. Does the patient have a sign on their door indicating respiratory isolation? [Yes/No]. If [Yes], skip to 6.
2. “Hello Mr/Ms [last_name], my name is (your name). I'm a medical student here at UAMS and am here to talk to you about the Covid vaccine. Do you have a few minutes to talk?” [Yes/No] If [Yes] skip to 4.

If [No] to 2:

1. “Thank you for your time. Here is some information about the Covid vaccine and a number to call if you are interested in scheduling your shot once you are ready.” [End]

If [Yes] to 2:

1. “Do you currently have symptoms from the COVID-19 virus or have a COVID test pending?” [Yes/No] If [Yes] skip to 6.
2. “Have you ever had a severe allergic reaction to vaccines in the past?” [Yes/No] If [No] skip to 7.
3. If [Yes] to respiratory precautions, symptoms or allergic reaction:

STOP - not eligible if patient is not being discharged from the ED, is symptomatic from COVID-19, or is on COVID precautions (sign on their door).

Skip to Demographics.

1. Thank you. Have you received your covid vaccine series yet?

[a. I have received all shots in my series (Pfizer, Moderna)]

[b. Yes, I received the single vaccination (Johnson&Johnson)]

[c. I have not received any shots yet but I have them scheduled.]

[d. The series is in process, I have received the first and am scheduled for the second.]

[e. I received the first but have not yet scheduled the second.]

[f. No, I have not received any covid vaccinations]

If [a, b]:

1. “That is great to hear. Do you have any questions about the Covid vaccine?”

Skip to 21 (Demographics).

If [e]:

1. “You're still needing part of your series. Do you need any help getting that scheduled?” [Yes/No]

If [c, d, e, f]:

1. “We are offering the Covid vaccine in the ER now - would you be willing to take it?” [Yes/No/Not sure]

If [c, d, e, f]:

1. “Do you have any concerns or questions about receiving the Covid vaccine?”

[Efficacy]

[Safety]

[Side effects]

[Don't believe it is necessary]

[Already had Covid-19 so don't need it]

[Cost or financial concerns]

[Other]

[No questions or concerns]

(Check all voiced questions/concerns)

Answer patient's questions or address concerns as you are able. If you have difficulty, or need assistance, please notify the attending for this patient. Any

notable interaction can be described here. __________________________________________

1. “Now that we have discussed your concerns, do you think you would be willing to receive the Covid vaccine today? There is no cost to you.” [Yes/No/Still unsure] If [Yes] skip to 14.

If [No/Still Unsure]

1. “Do you have other concerns regarding the vaccine?”

__________________________________________

Skip to 21 (Demographics).

If [Yes]

1. “That's great! If given the chance to choose, would you have a preference on which vaccine you receive? [Pfizer/Johnson&Johnson]
2. “Great! We can administer your shot today!”

[Yes - Vaccine given!!]

[No thanks - give handout] Skip to 21.

1. If the patient chooses J&J or does not have a preference, we will give J&J. We have this stocked in the main ED north side pyxis. Order the vaccine and ask the pt's physician to sign the order. The nurse can then be notified that the pt has a covid vaccine ordered. The vaccine card is provided.

(After vaccination)

1. Please notify the patient's nurse if the patient received a vaccine - they must be observed for 15 minutes for any severe allergic reaction!
2. Was the patient given the vaccine information sheet required by the CDC? [Yes/No]

Please give the patient the vaccine information sheet for the appropriate type of vaccine administered. This is Required!

Now is a good time to remind them that there can be occasional side effects such as arm soreness, body aches, fatigue, or fever for up to 48 hours after vaccination. This is normal and will go away. If these symptoms last longer than 48 hours, they should see their doctor.

1. Was the patient given their vaccine?

[Yes - given Pfizer]

[Yes - given J&J]

[No, pt refused]

[Pt agreed to schedule]

1. If the patient got the 1st dose of Pfizer, you must schedule their 2nd shot.

[2nd shot scheduled!]

[Pt unable to schedule 2nd shot today, information given.]

[N/a - this was pt’s second shot to complete the series.]

Please go to the Vaccine Clinic website to schedule. Patients do not come back to the ER for their 2nd shot.

Pfizer - 21 days (+/- 2 days) after first

1. “What is the gender you identify with?”

[Male]

[Female]

[Transgender, Male to Female]

[Transgender, Female to Male]

[None of the above]

[Prefer not to answer]

1. “What is your race?” (ok to read off the options)

[Black]

[White]

[Hispanic]

[Asian]

[American Indian/Alaska Native]

[Native Hawaiian/Other Pacific Islander]

[Multiple Races]

1. “What is your ethnicity?” (ok to read off the options)

[Hispanic or Latino]

[Not Hispanic or Latino]

1. “Thank you again for your time.”

[End]
