# Supplementary material for: Vaccine Acceptance During a Novel Student-led Emergency Department COVID-19 Vaccination Program
Source: West J Emerg Med. 2023 May 5;24(3):436–46. doi: 10.5811/westjem.58728 (PMC10284509; doi:10.5811/westjem.58728)
Supplement: Supplementary file 2 [file wjem-24-436-s002.docx]

**Appendix 2: Qualitative Interview Question Template for ED Covid Vaccine Initiative**

General questions

How much do you feel that giving covid vaccinations in the emergency department is supported by evidence?

What advantages or disadvantages are there for giving COVID vaccines in the ED?

How easy or hard is it to screen for and administer the covid vaccine in the ED? What has been your experience?

What barriers have you encountered to either the screening process or administering the vaccine?

What has made it easier to screen for or administer the vaccine?

Describe a case where the process worked. Now, how about a case where it didn’t work?

How could the process be improved?

How much do you feel continuing to administer covid vaccines in the ED is worthwhile or not worthwhile?

How much are you able to incorporate this into your workflow without disruption?

What is the perception among staff about giving covid vaccinations in the ED?

What is the perception among your leaders about giving covid vaccinations in the ED?

If we were going to make this process more automated, like through epic, what would be the best process? Who should do the screening?

How do you feel about co-administering flu and covid vaccines?

Specific student related questions:

How important is it to give covid vaccines in the ED?

How has assisting with this project advanced your education?

To what extent have you felt prepared or not prepared to screen and educate patients about the covid vaccine?

What was your experience with the patients?

How could the student process be improved to be more efficient or easier?
